# Access to Care for VA Dialysis Patients During Superstorm Sandy

**DOI:** 10.1177/2150132719863599

**Published:** 2019-07-26

**Authors:** Lilia R. Lukowsky, Aram Dobalian, David S. Goldfarb, Kamyar Kalantar-Zadeh, Claudia Der-Martirosian

**Affiliations:** 1 Veterans Emergency Management Evaluation Center (VEMEC), US Department of Veterans Affairs, North Hills, CA, USA; 2 University of Memphis School of Public Health, Memphis, TN, USA; 3 New York Harbor VA Healthcare System (NYHHS), New York, NY, USA; 4 NYU Langone Health, New York, NY, USA; 5 UCI School of Medicine, Orange, CA, USA

**Keywords:** veterans, vulnerable populations, natural disasters, Superstorm Sandy, dialysis

## Abstract

**Introduction:** This study examines the use of dialysis services by end-stage renal disease (ESRD) patients following the Superstorm Sandy–related, months-long closure of the New York campus of the US Department of Veterans Affairs (VA) New York Harbor VA Healthcare System (NYHHS, Manhattan VAMC). **Methods:** Outpatient visits, dialysis care, emergency department visits, and hospitalizations at VA and non-VA facilities for 47 Manhattan VAMC ESRD patients were examined 12 months pre- and post-Sandy using VA administrative and clinical data. **Results:** The Brooklyn campus of NYHHS, which is within ten miles of Manhattan VAMC, experienced the largest increase in the number of dialysis encounters after the closure. Dialysis encounters for VA patients also increased at non-VA facilities, rising on average, to 106 per month. For the James J Peters Bronx VAMC, the number of total dialysis encounters for Manhattan VAMC patients fluctuated between 39 and 43 per month, dropping to less than 30 after the Manhattan VAMC dialysis unit reopened. **Conclusion:** Manhattan VAMC ESRD patients used nearby alternate VA sites and non-VA clinics for their care during the closure of the Manhattan VAMC dialysis unit. The VA electronic health records played an important role in ensuring continuity of care for patients who exclusively used VAMC facilities post-Sandy because patient information was immediately accessible at other VA facilities. The events related to Superstorm Sandy highlight the need for dialysis providers to have a comprehensive disaster plan, including nearby alternate care sites that can increase service capacity when a dialysis facility is closed because of a disaster.

## Introduction

Patients with end-stage renal disease (ESRD) who undergo maintenance hemodialysis several times a week are at risk for increased morbidity and mortality when their care plans are interrupted for significant periods of time by disaster-related disruptions in equipment, electricity, water, communication, or transportation. For example, a month after Hurricane Katrina (2005), more than 50% of dialysis facilities in Louisiana remained closed due to major damage caused by the hurricane^1^; these closures contributed to an increase in renal-related hospitalizations in the hurricane-affected areas.^2^

During and immediately after Superstorm Sandy (October 29, 2012), 15 212 ESRD patients (1474 in Manhattan) sought dialysis care in 221 dialysis facilities located in the affected New York and New Jersey.^3^ On October 30, 2012, a total of 306 dialysis facilities in New York New Jersey were closed because of the storm.^4^ During the first week post-Sandy, 23% of ESRD patients who visited emergency departments (ED) received emergency dialysis.^3^ These service disruptions likely contributed to an increase in the 30-day mortality rate rising to 1.83% in Sandy-affected areas compared with 1.6% during the same month in the preceding year.^3^ About 59% of hemodialysis patients received early dialysis one to two days before Sandy, which was associated with lower odds of hospitalization during the first 10 days following Sandy, and lower 30-day mortality rate compared with ESRD patients who did not receive early dialysis.^3,5^

Those studies were conducted with non-Veteran dialysis patients in New York and New Jersey, and at present, no studies have examined the impact of Sandy on dialysis patients at the U.S. Department of Veterans Affairs (VA) facilities. Examining the impact of a major disaster like Sandy on VA ESRD patients is of interest because patients receiving care from a large, integrated health system like VA might be better able to continue to access care in the aftermath of such events compared with other ESRD patients. In general, VA patients tend to be older and have more physical and mental health diagnoses, and in particular, a higher prevalence of chronic kidney disease (CKD) compared with the general adult population.^6^ This may suggest that VA patients and specifically VA ESRD patients may be at greater risk for morbidity or mortality after a large disaster. According to Watnick et al^6^ more than 14 000 VA patients, who constituted about 50% of all ESRD VA patients in 2012, received hemodialysis through 69 hospital-based or free-standing outpatient VA dialysis clinics.

The dialysis unit at the New York, or Manhattan campus of the US Department of Veterans Affairs New York Harbor Healthcare System (NYHHS, Manhattan VAMC) was evacuated with the rest of the medical facility on October 28, 2012, one day prior to Sandy landfall,^7^ and did not reopen until mid-March 2013. All services were fully restored at the facility by mid-May 2013. The goal of this study is to examine the utilization of dialysis and other health care services by the Manhattan VAMC ESRD patients who were receiving maintenance hemodialysis at the facility 1 month prior to Sandy.

## Methods

### Cohort Description

A retrospective, longitudinal cohort study was conducted using VA administrative and clinical data. The Manhattan VAMC ESRD Sandy cohort was defined as patients who had received services at the facility’s dialysis unit 1 month before Sandy. The initial cohort included 118 patients who visited Manhattan VAMC at least once, 1 year prior to Sandy (October 29, 2011 to October 28, 2012) either for a dialysis treatment or who had a record of an ESRD-related diagnosis (ICD9: 585.5, 585.6, V56.0-V56.32, V45.11, V45.12), and were alive on October 29, 2012, the day Sandy made landfall in Manhattan.

From the initial study cohort, 47 patients were identified who received maintenance hemodialysis at the Manhattan VAMC 1 month prior to Sandy (September 29 to October 28, 2012). These 47 Manhattan VAMC patients constitute the ESRD Sandy cohort for this study. Using the VA electronic health records, all clinical encounters, including dialysis treatments, and inpatient, outpatient, and ED visits, were examined 1 year before and 1 year after Sandy (October 29, 2011 through October 28, 2013) for this cohort. This article assesses the patterns of utilization of dialysis and nondialysis VA services as well as VA-purchased services at non-VA facilities for this ESRD Sandy cohort 12 months pre- and 12 months post-Sandy. We hypothesize that post-Sandy, during Manhattan VAMC closure, majority of patients from the Manhattan VAMC ESRD cohort continued to utilize health care, including dialysis services at the VA facilities.

### Analysis

We compared the number of encounters per month per patient for 1 year before to 1 year after Sandy at neighboring VAMCs that offered outpatient dialysis services (Brooklyn and Bronx VAMCs) as well as at non-VA facilities located in the affected areas. The number and duration of inpatient stays in VA and non-VA facilities as well as the number of visits to EDs were also examined 1 year before and 1 year after Sandy. Paired *t* tests were used to analyze the differences between pre- and post-Sandy visits for each facility. All analyses were performed using SAS 9.4 and SAS Enterprise Guide 7.1 software packages (SAS Institute, Cary NC). This study was approved by the VA Greater Los Angeles Healthcare System Institutional Review Board.

## Results

### Patient Characteristics

Among the 47 ESRD veterans in the Sandy cohort, 45 were men, 15 were married, 13 were divorced or separated, 11 were never married, and 8 were widowed; 20 were older than 65 years (mean age 65 years; range 36-90 years); the average distance from patients’ home to Manhattan VAMC was 7 miles (Table 1). Out of 47 patients, 25 had diabetes, 39 had hypertension, 24 had ischemic heart disease, and 33 had at least 1 infection diagnosis during time of follow-up (Table 1). Four patients were receiving inpatient hemodialysis at the Manhattan VAMC prior to Sandy. When the Manhattan VAMC evacuated on October 28, 2012, three hospitalized ESRD patients were transferred to the Brooklyn VAMC, and 1 hospitalized ESRD patient was transferred to the Bronx VAMC. The median time passed between the last pre- and first post-Sandy dialysis was 5 days (excluding 9 patients with 15+ days, and 1 patient with no post-Sandy dialysis visits due to a missing date of first post-Sandy dialysis). Within 1 year after the hurricane, 8 ESRD Sandy patients had died, 1 had received a kidney transplant, and 5 had less than 60 recorded dialysis visits, which is substantially lower than the expected 150 annual dialysis visits for ESRD patients.

**Table 1.** Patient Characteristics for the Manhattan VAMC ESRD Sandy Cohort (N = 47).

Patient Demographics	n (%)
Male	45 (96)
Marital status	
Married	15 (32)
Never married	11 (23)
Divorced/Separated	13 (28)
Widowed	8 (17)
Age, years mean (range)	65 (36-90)
Age categories (years)	
18-44	3 (6)
45-64	24 (47)
65+	20 (47)
Comorbidities	
Heart failure	6 (13)
Dysthymia	13 (28)
Ischemic heart disease	24 (51)
Hypertension	39 (83)
Pulmonary vascular disease (PVD)	19 (40)
Anemia	43 (91)
Parathyroid conditions	7 (15)
Diabetes	25 (53)
Cancer	15 (32)
Other renal conditions	31 (66)
Hepatitis (any type)	16 (34)
HIV	5 (11)
Sepsis	10 (21)
Other infections	31 (66)
Mental health diagnoses	33 (70)
Opioid addiction	4 (9)
Other characteristics	
Distance to Manhattan VA from home address, miles, mean (range)	7 (0-16)
Time, days, passed between last pre- and first post-Sandy dialysis, median (range)	5 (0-15)
Post-Sandy follow-up	
Deaths	8 (17)
Incomplete encounters	5 (11)
Kidney transplant	1 (2)

Abbreviations: VAMC, Veterans Affairs Medical Center; ESRD, end-stage renal disease.

### Outpatient and Inpatient Encounters

Table 2 shows 1-year pre- and 1-year post-Sandy average numbers of encounters per patient as well as a total number of outpatient encounters, hospitalizations, ED visits, visits to non-VA facilities, and dialysis visits for the ESRD Sandy cohort. From October 29, 2011 through October 28, 2012 (1-year pre-Sandy) there were a total of 8136 outpatient encounters with an average of 173 per patient. Out of those visits, 383 were to non-VA facilities, 12 per patient on average, and 6183 (on average133 per patient) were for dialysis services. From October 29, 2012 through October 28, 2013 (1-year post-Sandy), the total number of outpatient encounters decreased to 6994 (corresponding to an average of 149 encounters per patient), non-VA facilities visits increased to 1895 (62 per patient), and dialysis visits decreased to 4977 (109 per patient). ED visits to VA and non-VA facilities decreased from 134 pre-Sandy to 59 post-Sandy, with respective averages of 3.4 and 1.5 visits per patient. We were able identity 3 patients who visited ED at the VA during the first month post-Sandy to receive emergency dialysis.

**Table 2.** Access to Care Before and After Hurricane Sandy for the Manhattan VAMC ESRD Sandy Cohort.^a^

	Pre-Sandy	Post-Sandy	*P*
Total outpatient visits per patient (total visits)^b^	173 (8136)	149 (6994)	.09
ED visits per patient (total ER visits)^c^	3.4 (134)	1.5 (59)	.002
ED visits leading to hospitalization per patient (total ED visits leading to hospitalization)^d^	1.6 (42)	0.8 (20)	.05
Total hospitalizations per patient (total hospitalizations)^e^	2.2 (88)	1.1 (42)	.001
Average days per hospitalization^e^	7	10	.8
Outpatient visits to non-VA facilities per patient (outpatient visits to non-VA facilities)^f^	12 (383)	62 (1895)	.001
Total dialysis visits per patient (dialysis visits)^b^	133 (6183)	109 (4977)	.03

Abbreviations: VAMC, Veterans Affairs Medical Center; ED, emergency department; ER, emergency room; ESRD, end-stage renal disease.

a Pre-Sandy: October 29, 2011 to October 28, 2012. Post-Sandy: October 29, 2012 to October 28, 2013.

b N Total/Pre-/Post-Sandy = 47.

c N Total = 39; N Pre-Sandy = 35; N Post-Sandy = 25.

d N Total = 27; N Pre-Sandy = 23; N Post-Sandy = 11.

e N Total = 39; N Pre-Sandy = 33; N Post-Sandy = 23.

f N Total = 31; N Pre-Sandy = 7; N Post-Sandy = 31.

### Dialysis and Outpatient Encounters by Facility

**Figure 1.**
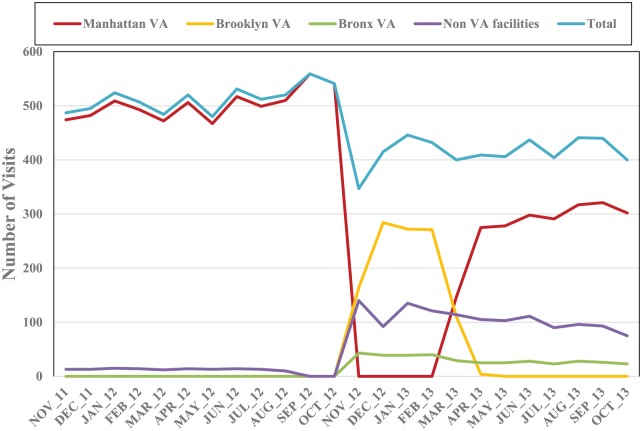
Monthly dialysis visits 1 year before and 1 year after Sandy by facility for the Manhattan VAMC ESRD Sandy Cohort (N = 47).

Figure 1 displays monthly dialysis visits for each facility from November 2011 through October 2013. One year before Sandy, at the Manhattan VAMC, there were an average of 502 dialysis visits per month (12 visits per patient). During the closure, no dialysis services were provided at the Manhattan VAMC; immediately after the Manhattan VAMC dialysis unit reopened (March 2013), the average number of visits per month increased to 7 per patient, and by April 2013 it returned to the pre-Sandy average of 12 visits per patient.

As shown in Figure 1, there were no dialysis visits for the ESRD Sandy cohort in Brooklyn or Bronx VAMCs during the 1 year before Sandy. However, the number of dialysis visits by Manhattan VAMC ESRD patients in Brooklyn VAMC increased from 0 to 164 in November 2012 during the closure, followed by 284, 272, and 271 visits in the following months, before dropping to 110 in March 2013 and to 4 in April 2013 after the Manhattan VAMC dialysis unit reopened. During the closure, the Brooklyn VAMC experienced the largest increase in the number of dialysis encounters.

The second largest increase in post-Sandy dialysis encounters for the Manhattan VAMC ESRD Sandy cohort occurred in non-VA facilities located in New York City. Pre-Sandy, there were no non-VA dialysis encounters by the study cohort. During November 2012, the number of dialysis visits to non-VA facilities increased to 140 as 20 patients used non-VA dialysis facilities at least once during that month. After the Manhattan VAMC dialysis unit reopened, the average number of dialysis visits to non-VA facilities decreased to 75 to 114 per month averaging to 83 visits by 10 patients monthly. For Bronx VAMC, the number of post-Sandy dialysis visits fluctuated between 39 and 43 per month, dropping to less than 30 (range: 23-29) after the Manhattan dialysis unit reopened. After the Manhattan VAMC campus completely reopened (by June 2013), 24 patients returned to the Manhattan VAMC dialysis unit, 10 continued using non-VA dialysis clinics, and 2 permanently switched to the Bronx VAMC.

**Figure 2.**
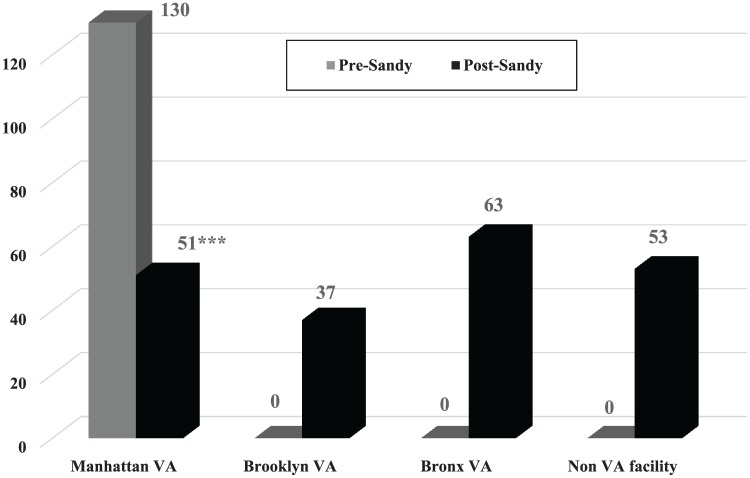
Average number of dialysis visits per-year per-patient by facility for the Manhattan VAMC ESRD Sandy Cohort (N = 47). Note: Pre-Sandy: October 29, 2011 to October 28, 2012. Post-Sandy: October 29, 2012 to October 28, 2013. Comparing pre- and post-Sandy for average number of visits for each facility: *P < .05; **P < .001; ***P < .0001.

**Figure 3.**
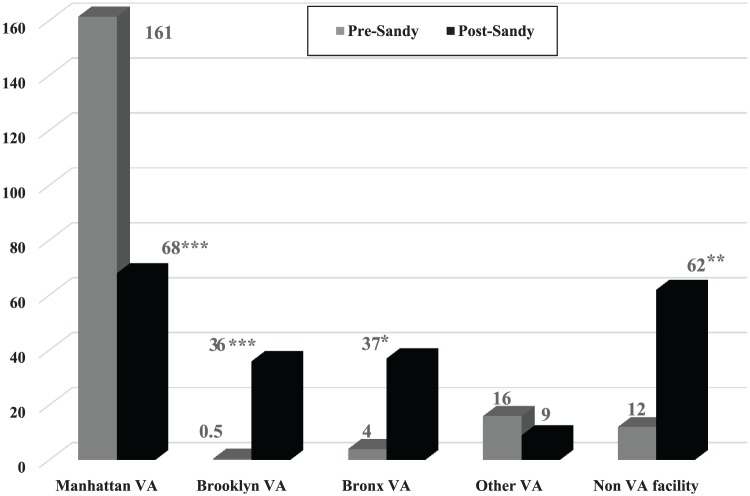
Average number of outpatient visits per-year per-patient by facility for the Manhattan VAMC ESRD Sandy Cohort (N = 47). Note: Pre-Sandy: October 29, 2011 to October 28, 2012. Post-Sandy: October 29, 2012 to October 28, 2013. Comparing pre- and post-Sandy for average number of visits for each facility: *P < .05; **P < .001; ***P < .0001.

The average number of annual dialysis encounters per patient decreased post-Sandy (130 vs 104; *P* = .008) as did the average annual number of total outpatient encounters per patient (173 vs 149; *P* = .09) (see Table 2, Figures 2 and 3). At the Manhattan VAMC, the average number of annual dialysis encounters decreased from 130 to 51 (*P* < .0001) per patient (see Figure 2). At the same time, the Brooklyn (0 to 37) and Bronx VAMCs (0 to 63) and the non-VA facilities in NYC (0 to 53) all experienced an increase in dialysis encounters. There were similar patterns for the total outpatient encounters for these facilities (see Figure 3).

## Discussion

Our study shows that during the months that the Manhattan VAMC was closed, VA maintenance hemodialysis patients received medical care from both alternate VA and non-VA facilities. After the initial interruption of services in the first week following the storm, most ESRD VA patients were able to resume their regular treatment schedule at an alternate site.

Previous studies have shown that patient populations with special needs are at a greater risk of displacement, illness, or even death during natural disasters. Other studies have shown that households which have members with disabilities are not better prepared for disasters despite having greater vulnerability.^8-11^ During Superstorm Sandy, 6300 patients at 37 health care facilities in New York City were evacuated because of extensive flood damage, creating a surge of new patients to already overcrowded EDs at nearby facilities.^7,12-18^ Additionally, many health care facilities, including dialysis clinics, in the affected areas closed, interrupting services for ESRD patients. The majority of ESRD patients missed at least one dialysis session following Sandy, and some received emergency dialysis.^3-5,16,19,20^ There were delays accessing ESRD patients’ treatment records and Sandy-affected medical facilities experienced shortages of trained medical personnel and equipment. Many ESRD patients in lower Manhattan were forced to seek dialysis treatment at a limited number of emergency and dialysis facilities that were still open during Sandy.^4,5,19-21^

For the ESRD Sandy cohort, it took a median of 5 days to resume dialysis treatments after Sandy, an increase of 3 days from the usual 2-day dialysis-free interval. All Manhattan VA ESRD patients, except the inpatient transfers, missed at least one dialysis treatment.^22^ This was considerable higher than a 25% of reported ESRD patients who missed at least 1 dialysis session following Sandy.^21^ In our study cohort, we identified 3 patients who visited EDs to receive dialysis during the first month post-Sandy. Once transportation services resumed, many of the ESRD Sandy patients accessed care at the Brooklyn VAMC where Manhattan VAMC personnel set up a small unit to provide maintenance hemodialysis. Since the Brooklyn VAMC is part of the VA health system, the largest integrated health care system in the United States, the Manhattan VAMC patients’ health records were accessible by the medical personnel at Brooklyn VAMC as well as at Bronx VAMC.^22^ However, there were problems reported at non-VA hospitals and dialysis clinics immediately after Sandy.^4,19-21^ A retrospective survey of 14 hospitals in Brooklyn by Lin et al^20^ reported 30% to 150% increased surge capacity of dialysis units post-Sandy.^20^ One of the major problems reported was lack of essential information by transient patients about dialysis prescription and hepatitis status, which resulted in increased wait time for blood tests, contacting patients’ home facilities (many closed), and disinfecting equipment after it was used by patients with unknown hepatitis status. By sharing patient medical records between VAMCs, VA patients receiving dialysis at multiple VAMC facilities avoided these difficulties.

In addition to shared electronic health records, VA ESRD patients benefited from the VA’s ability to shift equipment and medical personnel from the inaccessible Manhattan VAMC to the Brooklyn and Bronx VAMCs. Out of 14.5 staff (2.5 medical doctors, 1 head nurse, 7 registered nurses, 1 licensed practical nurse, 1 nurse practitioner, 2 technicians) of Manhattan VAMC dialysis unit, 14 full-time members temporarily relocated to Brooklyn VAMC, a part-time physician relocated to Bronx. This shift underscores the potential advantages of a health system in providing post-disaster surge capacity in order to maintain continuity of care after major disasters, although it should be noted that VA still found it necessary to purchase care from some non-VA facilities, at least partly to permit easier geographic access to regular dialysis treatment for some veterans.

We found a 16% decrease in total post-Sandy encounters for the ESRD Sandy cohort, including a decrease in both VA ED visits and VA hospitalizations. These findings differ from previously reported studies on non-veteran ESRD patients that found an increase in ED visits and hospitalizations immediately following Sandy.^2,3,20^ Our observed decrease in post-Sandy encounters may be due to the differences in study designs. Unlike previous studies, this study used a retrospective, longitudinal cohort design where the trajectory of health care services used for the study cohort was tracked 1 year post-Sandy. The decrease in the number of patients and patient encounters is not surprising due to deaths or loss to follow-up (ie, moving out of the area, switching health care providers from VA to Medicare, or receiving a kidney transplant). Although the number of post-Sandy VA hospitalizations decreased, the average number of days spent in the hospital increased by 3 days, perhaps related to a need to stabilize patients following disruptions in care during and immediately after Sandy.

### Limitations

The study has limitations. VA ESRD patients who did not use VA dialysis services either directly or through VA-purchased care were not included in this study since we did not have access to Medicare data. Additionally, we only accessed VA encounters in the Sandy-affected region (New York, New Jersey, Pennsylvania), and therefore were not able to assess use of VA services outside of the affected area. While we have incomplete information about outpatient encounters for 5 ESRD Sandy patients, after conducting a sensitivity analysis, which assumed the same number of dialysis visits as the year prior to Sandy, post-Sandy health care utilization patterns did not change.

## Conclusion

We found that the majority of ESRD VA patients receiving maintenance hemodialysis at the Manhattan VAMC dialysis unit accessed dialysis and other services at neighboring VA and non-VA medical facilities within 5 days after Hurricane Sandy. During the several months that the Manhattan VAMC remained closed, ESRD VA patients received dialysis treatments at several medical facilities, including the Brooklyn VAMC (where medical personnel from the Manhattan dialysis unit temporarily relocated), the Bronx VAMC, and non-VA facilities that were covered by VA-purchased care.

Superstorm Sandy highlights the need for dialysis providers to have a comprehensive disaster plan that includes nearby alternate care sites that can increase service capacity when a dialysis facility is closed because of a disaster. The VA electronic health records ensured continuity of care at other VAMCs because patient information was immediately accessible at those VA facilities. These factors likely limited the impact of Sandy on Veterans’ care and reduced the potentially severe complications that otherwise might have occurred due to interruptions in care from the hurricane.
